# Dynamic Development of Fecal Microbiome During the Progression of Diabetes Mellitus in Zucker Diabetic Fatty Rats

**DOI:** 10.3389/fmicb.2019.00232

**Published:** 2019-02-14

**Authors:** Wen Zhou, Huiying Xu, Libin Zhan, Xiaoguang Lu, Lijing Zhang

**Affiliations:** ^1^Modern Research Laboratory of Spleen Visceral Manifestations Theory, Basic Medical College, Nanjing University of Chinese Medicine, Nanjing, China; ^2^Department of Emergency Medicine, Zhongshan Hospital, Dalian University, Dalian, China

**Keywords:** 16S gene sequencing, fecal microbiome, type 2 diabetes mellitus, gut microbiota, time series, rat microbiome

## Abstract

**Background:** Although substantial efforts have been made to link the gut microbiota to type 2 diabetes, dynamic changes in the fecal microbiome under the pathological conditions of diabetes have not been investigated.

**Methods:** Four male Zucker diabetic fatty (ZDF) rats received Purina 5008 chow [protein = 23.6%, Nitrogen-Free Extract (by difference) = 50.3%, fiber (crude) = 3.3%, ash = 6.1%, fat (ether extract) = 6.7%, and fat (acid hydrolysis) = 8.1%] for 8 weeks. A total of 32 stool samples were collected from weeks 8 to 15 in four rats. To decipher the microbial populations in these samples, we used a 16S rRNA gene sequencing approach.

**Results:** Microbiome analysis showed that the changes in the fecal microbiome were associated with age and disease progression. In all the stages from 8 to 15 weeks, phyla *Firmicutes*, *Bacteroidetes*, *Actinobacteria*, and *Proteobacteria* primarily dominated the fecal microbiome of the rats. Although *Lactobacillus* and *Turicibacter* were the predominant genera in 8- to 10-week-old rats, *Bifidobacterium*, *Lactobacillus*, *Ruminococcus*, and *Allobaculum* were the most abundant genera in 15-week-old rats. Of interest, compared to the earlier weeks, relatively greater diversity (at the genus level) was observed at 10 weeks of age. Although the microbiome of 12-week-old rats had the highest diversity, the diversity in 13–15-week-old rats was reduced. Spearman’s correlation analysis showed that F/B was negatively correlated with age. Random blood glucose was negatively correlated with *Lactobacillus* and *Turicibacter* but positively correlated with *Ruminococcus* and *Allobaculum* and Simpson’s diversity index.

**Conclusion:** We demonstrated the time-dependent alterations of the abundance and diversity of the fecal microbiome during the progression of diabetes in ZDF rats. At the genus level, dynamic changes were observed. We believe that this work will enhance our understanding of fecal microbiome development in ZDF rats and help to further analyze the role of the microbiome in metabolic diseases. Furthermore, our work may also provide an effective strategy for the clinical treatment of diabetes through microbial intervention.

## Introduction

Type 2 diabetes mellitus (T2DM) is currently the most prevalent metabolic disease in the world and is characterized by insulin resistance, with an initial increase in insulin secretion, but subsequent beta cell death and insulin insufficiency over time. According to the International Diabetes Federation, T2DM will affect 693 million people worldwide by 2045 (Cho et al., 2018). T2DM is a multifactorial disorder, with pathogenic contributions from genetic, environmental, and lifestyle factors (Mengual et al., 2010; Saxena et al., 2012). The gut microbiota has increasingly been recognized as a key contributor to T2DM, and T2DM can be linked to dysbiosis of the intestinal microbiota (Cox et al., 2014; Forslund et al., 2015; Yano et al., 2015). Two independent studies based on fecal samples from European and Chinese populations showed increased abundances of opportunistically pathogenic *Clostridium* species and decreased abundances of butyrate-producing *Roseburia*, *Faecalibacterium*, and *Eubacterium* species associated with T2DM patients (Qin et al., 2012; Karlsson et al., 2013). Karlsson et al. (2013) also found that increased abundances of *Lactobacillus gasseri* and *Streptococcus mutans* can predict insulin resistance, while Qin et al. (2012) found enrichment in *Escherichia coli* associated with current T2DM patients. Some studies have also found that pro-inflammatory bacteria such as *Ruminococcus* *gnavus* and *Bacteroides* spp. are more common in the feces of T2DM patients (Everard and Cani, 2013). Numerous studies have shown significant changes in the composition and diversity of the fecal microflora under conditions of diabetes. Studies also speculate that changes in the composition and diversity of feces can determine the prognosis and severity of T2DM. Our understanding of the relevance of the microbiome in metabolic diseases might be enhanced by systematically assessing the role of the fecal microbiota in disease performance and its control.

An understanding of the fecal microbiome in T2DM has recently arisen by analyzing microbial populations found in fecal samples at a certain point in time. Although such assessments of the fecal microbiome composition and diversity in T2DM are valuable, they are time-limited and do not reflect the dynamic changes of microbial flora in the progression of T2DM. Several studies have reported that the fecal microbiome differs at different times during the progression of T2DM (Horie et al., 2017; Liu et al., 2017). Therefore, more work needs to be done to determine the role of the fecal microbiome diversity and composition and their association with T2DM. Due to ethical issues and the availability of a limited number of samples, analysis of the fecal microbiome and its role in the disease pathogenesis of diabetes in humans is limited. Thus, to establish the diabetic fecal microbiome, small animal models can be used. In these models, fecal samples can be conveniently collected, thereby allowing for investigation of the microbiome contribution in T2DM. In fact, to understand the role of the microbiome in T2DM, many animal models have been widely used (Bagarolli et al., 2017; Bindels et al., 2017; Caparros-Martin et al., 2017). In addition, evidence emerging from animal models shows that many of the symptoms associated with diabetic syndrome and insulin sensitivity may be improved through replenishing probiotics (*Lactobacillus rhamnosus*, *Lactobacillus acidophilus*, and *Bifidobacterium*) and butyric-acid producing bacteria *Clostridium butyricum* (Bagarolli et al., 2017; Jia et al., 2017). Although some studies have used rat models to elucidate the microbiome’s role in T2DM (Goldsmith et al., 2017; Kim et al., 2017), in the field of T2DM, one of the major unanswered questions is whether the microbiome can be utilized to alleviate diabetic pathologies.

Currently, only a few studies have examined details about the compositional dynamics of the diabetic microbiome (Horie et al., 2017; Liu et al., 2017). Since most of these studies were conducted at some point in the course of T2DM development, they do not provide an insight into the development of the diabetic fecal microbiome. Using animal models might establish a better understanding of the fecal microbiome in the progression of T2DM, and such knowledge can enhance our understanding of the microbiome effects on T2DM. ZDF rats with a missense mutation (fatty, fa) in the leptin receptor gene can develop obesity, insulin resistance, and T2DM (Phillips et al., 1996; Yamashita et al., 1997; Da Silva et al., 1998; Yokoi et al., 2013). Male ZDF rats exhibit an age-dependent diabetic phenotype that develops hyperglycemia at 8 weeks of age and the blood glucose level remains high throughout its lifespan (De Lemos et al., 2007). Due to these characteristics, ZDF rats are an attractive experimental model for this study. In this study, we monitored body weight, food intake, water intake, rectal temperature, RBG, OGTT, and the fecal microbiome from 8 to 15 weeks of age in ZDF rats. We analyzed the fecal microbiome at different time points in diabetic rats and tracked changes in microbial diversity. A deep sequencing of 16S rRNA genes amplified from genomic DNA isolated from the rat feces was used. To this end, we also performed non-parametric Spearman’s correlation analysis to evaluate associations between physiological characteristics and the microbiome in ZDF rats.

## Materials and Methods

### Experimental Design

This study was done longitudinally and its primary purpose was to understand the changes in fecal microbiome composition during diabetes progression in four ZDF rats. We studied the microbiome from week 8 onward to week 15 at 1-week intervals. Studies were performed using ZDF rats as they have been shown to exhibit hyperinsulinemia and hyperglycemia (De Lemos et al., 2007) and are thus a good model of T2DM.

### Ethics Statement

In the present study, the animal experiments used rats and were approved by the Animal Ethics Committee of Nanjing University of Chinese Medicine (Approval No. ACU170606). All animal experiments were conducted in accordance with the National Institutes of Health Guide for the Care and Use of Laboratory Animals at Nanjing University of Chinese Medicine (Nanjing, China).

### Animal

Four male 6-week-old ZDF rats were purchased from Vital River Laboratories (Beijing, China) and housed in a specific pathogen-free animal experimental center in Nanjing University of Chinese Medicine. Animals were fed autoclaved Purina 5008 chow [protein = 23.6%, Nitrogen-Free Extract (by difference) = 50.3%, fiber (crude) = 3.3%, ash = 6.1%, fat (ether extract) = 6.7%, and fat (acid hydrolysis) = 8.1%; Vital River Laboratories, Beijing, China], had free access to autoclaved water, and housed at 24°C ± 2°C, humidity 65% ± 5%, with a 12 h light-dark cycle. During the trial, body weight, food and water intake, and rectal temperature were measured daily. All rats were in one group and housed in one cage during the study.

### Random Blood Glucose Test

Random blood glucose was measured weekly to examine the progression of diabetes in ZDF rats. Glucose levels in tail blood samples were measured from weeks 8 to 15 using a glucometer (CareSens, I-SENS, Anyang, South Korea). The rats were not fasted for RBG tests.

### Oral Glucose Tolerance Test

Zucker diabetic fatty rats were fasted for 14 h (overnight) and then the OGTT was performed with a glucose solution in saline at 2 g/kg. Tail blood was sampled at 0, 30, 60, and 120 min after glucose administration. Glucose levels were determined immediately with a glucometer (CareSens, I-SENS).

### Stool Sample Collection and DNA Extraction

One fresh fecal sample was collected directly from the anus into a sterile tube from each rat weekly, avoiding contact with rat skin or urine (see Supplementary Table S1). A total of 32 stool samples were collected from weeks 8 to 15 in four ZDF rats and stored at −80°C prior to processing. Bacterial DNA was extracted from feces using the Fast DNA SPIN extraction kit (MP Biomedicals, Santa Ana, CA, United States) according to the manufacturer’s instructions and stored at −20°C before further analysis. The quantity and quality of extracted DNA were measured using a NanoDrop ND-1000 spectrophotometer (Thermo Fisher Scientific, Waltham, MA, United States) and agarose gel electrophoresis, respectively.

### 16S rRNA Amplification and Sequencing

PCR amplification of the bacterial 16S rRNA genes (V3–V4 region) was carried out using forward primer 338F (5′-ACTCCTACGGGAGGCAGCA-3′) and reverse primer 806R (5′-GGACTACHVGGGTWTCTAAT-3′). Sample-specific 7-bp barcodes were incorporated into the primers for multiplex sequencing. PCR components contained 5 μl Q5 reaction buffer (5×), 5 μl Q5 High-Fidelity GC buffer (5×), 0.25 μl Q5 High-Fidelity DNA polymerase (5 U/μl), 2 μl dNTPs (2.5 mM), 1 μl of each forward and reverse primers (10 μM), 2 μl DNA template, and 8.75 μl ddH_2_O. Thermal cycling included initial denaturation for 2 min at 98°C, followed by 25 cycles including denaturation for 15 s at 98°C, annealing for 30 s at 55°C, and extension for 30 s at 72°C, and a final extension of 5 min at 72°C. PCR amplicons were purified using Agencourt AMPure Beads (Beckman Coulter, Indianapolis, IN, United States) and quantified with the PicoGreen dsDNA Assay Kit (Invitrogen, Carlsbad, CA, United States). After the individual quantification step, amplicons were combined in equal amounts and subjected to 2 × 300 bp sequencing of the end using the Illumina MiSeq platform and the MiSeq kit v3 from Shanghai Personal Biotechnology Co., Ltd. (Shanghai, China). Sequencing data were processed using a quantitative analysis of microbial ecology (QIIME, v1.8.0). In brief, original sequencing reads that perfectly matched the barcode were assigned to the corresponding samples and identified as valid sequences. Low-quality sequences (Gill et al., 2006; Chen and Jiang, 2014) were filtered by the following criteria: sequences <150 bp in length, sequences with average Phred scores <20, sequences containing indefinite bases, and sequences containing single nucleotide repeats of >8 bp. Paired-end reads were assembled using FLASH (Magoc and Salzberg, 2011). After chimera detection, the remaining high-quality sequences were clustered into OTUs with 97% sequence identity by UCLUST (Edgar, 2010). The default parameters were used to select the representative sequence from each OTU. Using the best hits (Altschul et al., 1997), OTU taxonomy classification was performed by a BLAST search on the representative set of sequences against the Greengenes database (Desantis et al., 2006). The abundance of each OTU in each sample and the taxonomy of these OTUs were recorded by generating an OTU table. OTUs with a total content of less than 0.001% in all samples were discarded. To minimize the difference in the depth of sequencing across samples, the average analysis of 100 evenly resampled OTU subsets under the 90% of the minimum sequencing depth was performed to generate an average, rounded dilution OTU table.

### Bioinformatics Analysis

Sequencing data were evaluated using the QIIME and R software packages (v3.2.0). The OTU table in QIIME was used to calculate the α diversity index of the OTU level, such as the Shannon diversity index and the SDI. Principal weighted UniFrac distance metrics (Lozupone and Knight, 2005) were used for principal coordinate analysis (PCoA). Diversity was assessed using the Simpson Diversity Index (SDI) by calculating “inverse” (1/λ) and “complement” (1-λ) SDI. Higher SDI values indicated higher microbial diversity. Based on the occurrence of OTUs across samples, a petal diagram was created to visualize the shared and unique OTUs among samples or groups by the R package “Venn Diagram.” Metastats (White et al., 2009) was used to statistically compare the abundance of taxa at the level of phylum and genus among samples or groups.

### Statistical Analysis

The physiological characteristics data of the ZDF rats are presented as mean ± SD. Statistical analyses among different ages were performed by repeated ANOVA, followed by Tukey’s honestly significant difference or Dunnett’s *post hoc* test with SPSS 19.0 (IBM, Chicago, IL, United States), considering a *P*-value ≤ 0.05 as statistically significant. Correlations between physiological characteristics data and either *F*/*B* ratio or genus were tested by Spearman’s correlation analysis using Prism 5 (GraphPad, La Jolla, CA, United States).

### Sequence Accession Numbers

The datasets generated in this study are available through the NCBI Sequence Read Archive (accession number SRP148630).

## Results

### Physiological Characteristics of ZDF Rats From 8 to 15 Weeks

**FIGURE 1:**
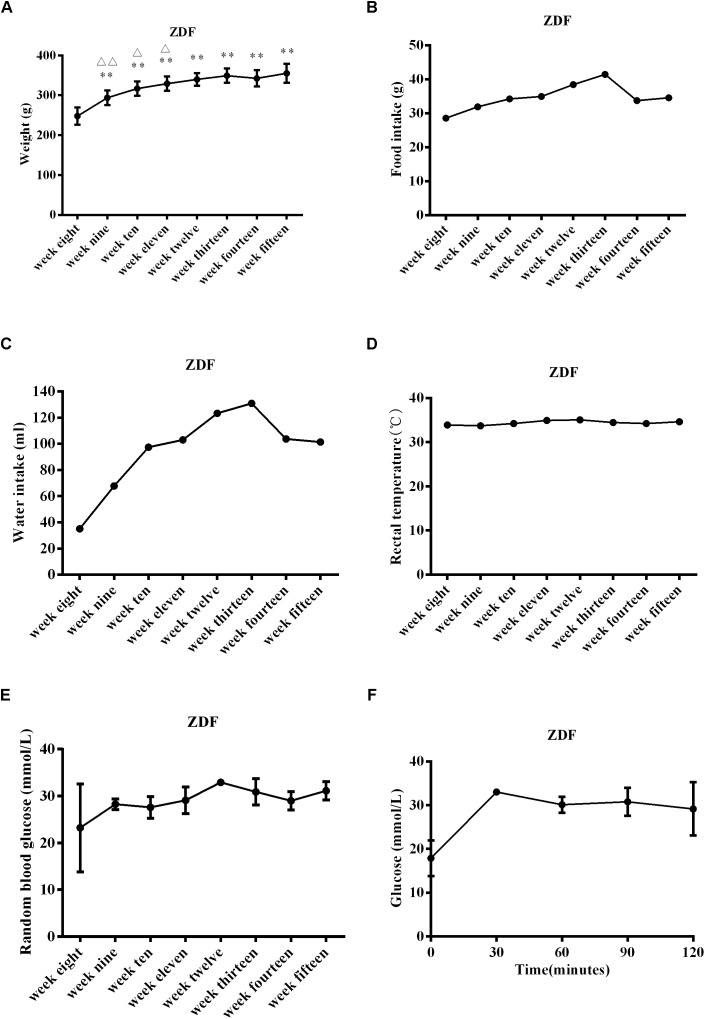
Physiological characteristics in ZDF rats ranging from 8 to 15 weeks old. **(A)** Body weight gain in ZDF rats. **(B)** Food intake changes in ZDF rats. **(C)** Water intake changes in ZDF rats. **(D)** Rectal temperature changes in ZDF rats. **(E)** Random blood glucose levels in ZDF rats. **(F)** Oral glucose tolerance test measured at week 14 in ZDF rats. Data are expressed as mean ± SEM. *N* = 4 in individual groups; data were analyzed by repeated ANOVA: compared with week 8, ^∗^*p* < 0.05, ^∗∗^*p* < 0.01, ^∗∗∗^*p* < 0.001; compared with the previous week, ^Δ^*p* < 0.05, ^ΔΔ^*p* < 0.01, ^ΔΔΔ^*p* < 0.001.

Zucker diabetic fatty rats gained significantly more weight from 9 to 15 weeks of age compared to weights at 8 weeks of age (*P* < 0.01 at weeks 9–15, Figure 1A). Compared with the previous week, ZDF rats gained significantly more weight at 9, 10, and 11 weeks of age (*P* < 0.01 at week 9, *P* < 0.05 at weeks 10–11, Figure 1A). ZDF rats generally experienced an upward trend in food and water intake from 8 to 15 weeks (Figure 1B,C), but the rectal temperature remained stable (Figure 1D). Insulin sensitivity was assessed by measuring RBG levels and by the OGTT at week 14. RBG levels in 8-week-old ZDF rats reached diabetes status (Figure 1E). The OGTT showed that the blood glucose level reached the highest value at 30 min, and then gradually decreased, but still could not recover to the initial value at 120 min (Figure 1F). These findings are consistent with previous reports (Jourdan et al., 2013; Wessels et al., 2015; Van Bree et al., 2016; Szokol et al., 2017) and indicate that the ZDF rats presented with pathological conditions of diabetes. The disease was generally aggravated with age, glucose tolerance was impaired, and insulin sensitivity was reduced.

### Developing T2DM Harbors Temporally Dynamic Microbial Diversity

**FIGURE 2:**
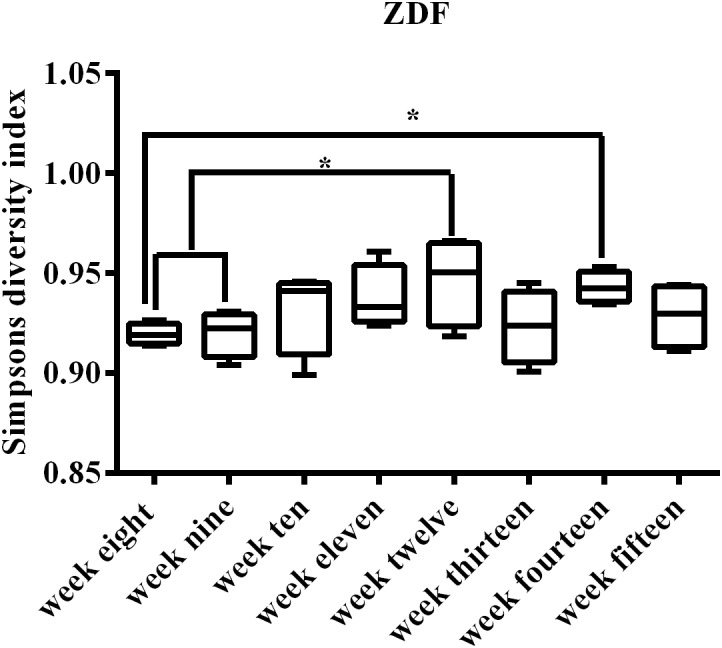
Simpson’s diversity index for all 32 samples representing the weeks of development T2DM. Early weeks (8–9) have lower diversity, and the increase in diversity at 10 weeks of age can be clearly visualized from the box plots. Median (line within the box) and minimum and maximum values (whiskers) are illustrated by box and whisker plots. Compared within the weeks, ^∗^*p* < 0.05.

The progression of diabetes may be associated with microbiome dynamic changes; thus, we tracked the fecal microbiome changes in rats from 8 weeks of age until 15 weeks of age. Of note, we did not include rats older than 15 weeks of age in this work because published literature suggests that male ZDF rats exhibit significant diabetic complications at 15 weeks of age (Gu et al., 2017). A total of 1,944,426 16S rRNA (V3–V4 region) reads were obtained, averaging 60,763 reads per sample. Reads were undertaken to generate a total of 44,613 OTUs, which could be further grouped into ∼315 unique OTUs. Collectively, these sequences represented 247 unique genera. The average Shannon Diversity Index for all time points ranged from 4.87 to 6.28, with an average of 5.52 (confidence intervals for all SDI values are provided in Supplementary Table S2). Between Shannon and Simpson’s diversity indices, there was a consistent trend. Using the SDI could clearly visualize the trends (Figure 2). SDI described an increase in diversity from 9 to 12 weeks in ZDF rats, with the highest diversity observed at 12 weeks of age, followed by a slight decrease at 13–15 weeks of age. This trend was repeatable using the inverse SDI (Supplementary Figure S1A). The median and inter-quartile range (IQR) are provided in Supplementary Figure S1B.

**FIGURE 3:**
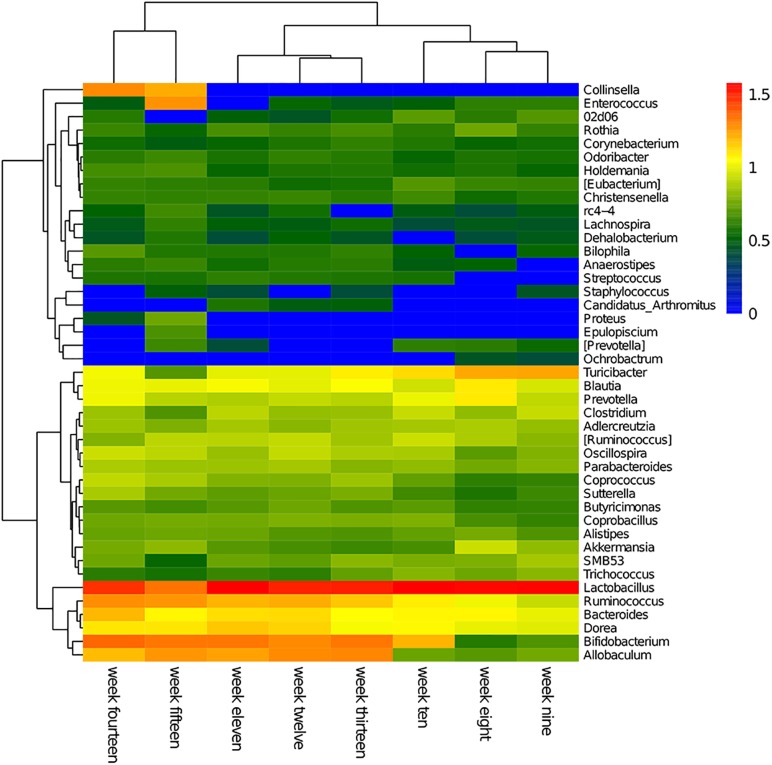
Hierarchical clustering using Euclidean distances was used to construct an inter-week genus-level heatmap. In the figure, red represents high abundance and blue represents low abundance.

The cluster heatmap for each genus per week is shown in Figure 3. The abundance levels of each genus in the cluster heatmap revealed the weekly dominant genera. Overall, the fecal microbiome consisted of unique genera that can reflect the diversity and dynamic changes of a microbial population.

### Identification of Core Microbial Communities in the Diabetic Stage of ZDF Rats

**FIGURE 4:**
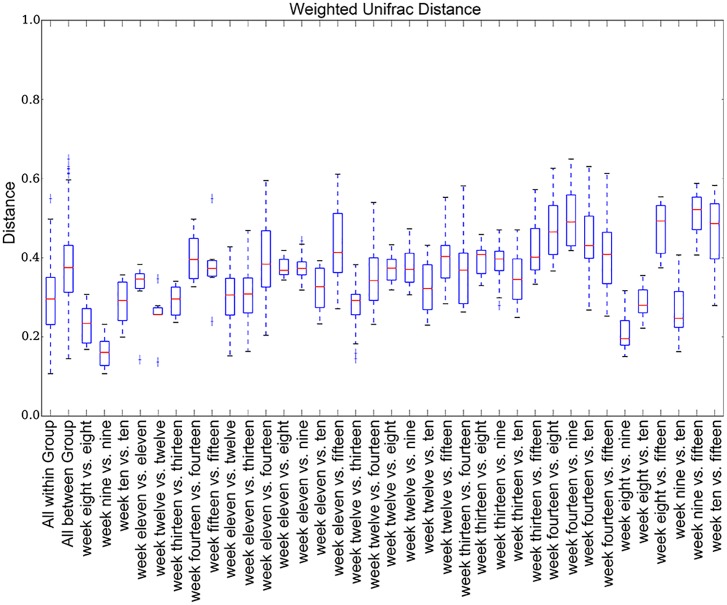
Weighted UniFrac distance box plots. The inter-week weighted UniFrac distances are longer than the intra-week distances.

The number of rats per week was 4 (see Supplementary Table S3 for the number of each sample). We plotted the weighted UniFrac distances for all weeks (Figure 4) to compare abundance across weeks. Inter-week weighted UniFrac distances were longer than intra-week weighted UniFrac distances.

**FIGURE 5:**
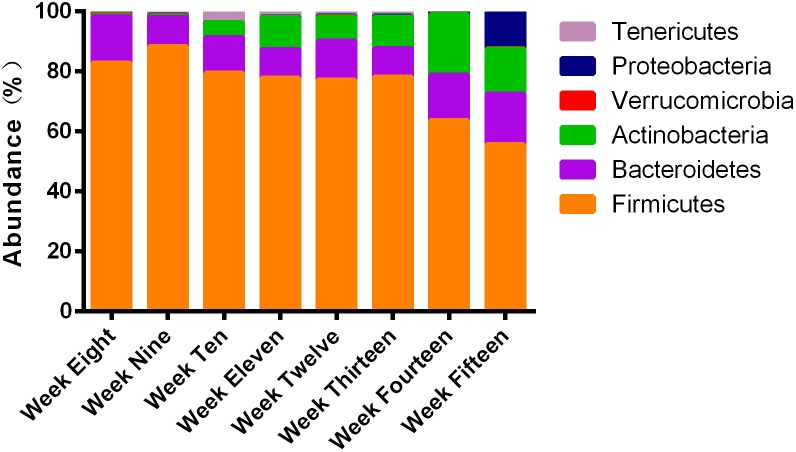
Four phyla: *Firmicutes*, *Bacteroidetes*, *Actinobacteria*, and *Proteobacteria* dominated the majority of ∼315 OTUs. 16S rRNA gene sequences were used to establish the identities. Different proportions of phyla can be seen at different stages of diabetes development in ZDF rats. More than 90% of the reads belonged to these four phyla.

At the phyla level, compared to the relative percent abundance, more than 90% of the microbial population in ZDF rats from weeks 8 to 15 consisted of the phyla *Firmicutes*, *Bacteroidetes*, *Actinobacteria*, and *Proteobacteria*. During 8–15 weeks of age, the most abundant phylum was *Firmicutes* (Figure 5). At 8–9 weeks of age, the predominant phyla were *Firmicutes* and *Bacteroidetes*. *Actinobacteria* gradually increased from the 10th week of age until the 15th week of age. *Proteobacteria* increased significantly in ZDF rats at 15 weeks of age compared to other ages. We have plotted the mean abundance measure along with the standard error for individual phyla (Supplementary Figure S2). It is worth noting that the percent abundance of different phyla varied at every week, thereby suggesting a dynamic microbial ecosystem in ZDF rats.

### Grouping of Microbial Abundance in the Feces of ZDF Rats Shows Temporal Signatures

**FIGURE 6:**
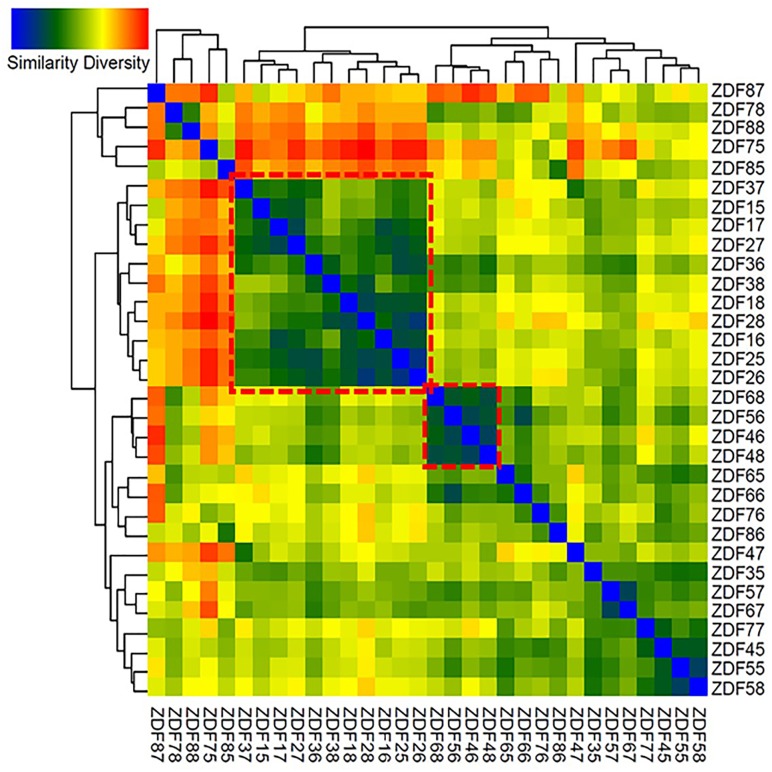
Heatmap generated of the 32 samples. Clustering similarity based on Weighted UniFrac distance matrix data was performed to construct the heatmap; blue in the graph represents high similarity and red represents low similarity. ZDF15–18 represents fecal samples from 8-week-old ZDF rats, ZDF25–28 represents fecal samples from 9-week-old ZDF rats, ZDF35–38 represents fecal samples from 10-week-old ZDF rats, ZDF45–48 represents fecal samples from 11-week-old ZDF rats, ZDF55–58 represents fecal samples from 12-week-old ZDF rats, ZDF65–68 represents fecal samples from 13-week-old ZDF rats, ZDF75–78 represents fecal samples from 14-week-old ZDF rats, and ZDF85–88 represents fecal samples from 15-week old ZDF rats.

To identify differences and similarities between the microbial populations in different samples, cluster analyses based on weighted UniFrac distances (Lozupone and Knight, 2005) were carried out. These analyses revealed that weeks 8–10 and 11–13 showed mixed effects and formed two distinct clusters (Figure 6). Some samples clustered with other samples from the same week, thus exhibiting high specificity (samples from week 12). Samples from other weeks either clustered non-specifically with other samples or clustered with the nearest neighboring time point (weeks 11–12 and 13–14).

**FIGURE 7:**
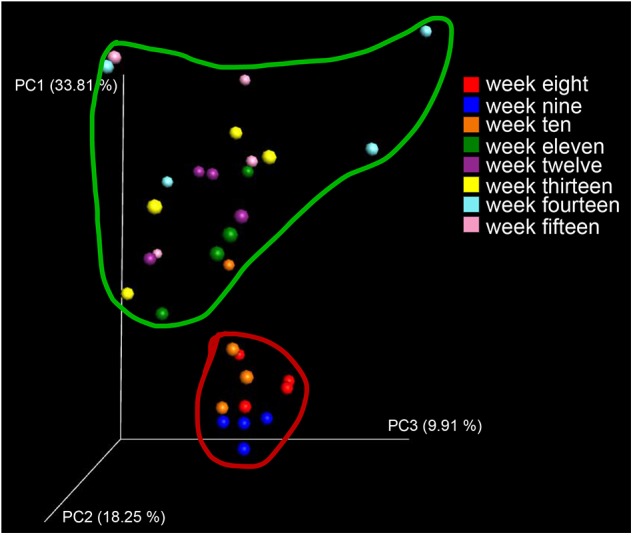
Diversity and distribution of OTUs at different stages of diabetes development. As a measure of beta diversity, PCoA of weighted UniFrac distances (between samples diversity): samples from week 8 (red dots), week 9 (blue dots), and week 10 (brown dots) grouped together into a cluster (when viewed in 3D along the PC3 axis).

**FIGURE 8:**
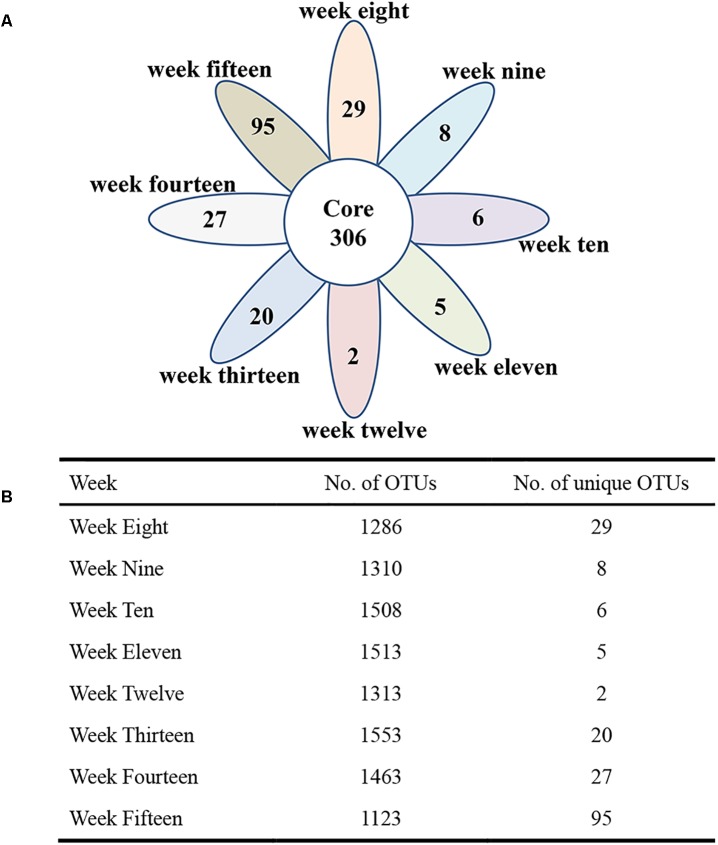
The petal diagram reveals common and unique genera associated with different stages of diabetes development. Different colors represent different modules. **(A)** The petal diagram (nodes) at the center of the petal diagram (∼306) is shared by all weeks. **(B)** The total number of OTUs and the number of unique OTUs are shown in the table.

To visualize whether the samples could form distinct clusters, weighted UniFrac distances were used for the principal coordinate analysis (PCoA). Whereas samples from week 8 (red circle), week 9 (blue circle), and week 10 (brown circle) grouped together in a cluster (along the PC3 axis), the remaining samples (weeks 11–15) grouped into a large cluster (Figure 7). To display the number of common and unique OTUs presented in each group during the progression of diabetes, a petal diagram was constructed (Figure 8A). It revealed that among all the weeks, ∼306 OTUs were shared. It enabled us to more clearly visualize those OTUs that were distinct for each time scale [ranges from 2 (week 12) to 95 (week 15)] (Figure 8B). The dominant phyla of these unique OTUs were *Firmicutes*, *Bacteroidetes*, *Actinobacteria*, and *Proteobacteria*.

### Microbial Diversity Initially Increases and Then Decreases With Age and Disease Progression in ZDF Rats

**FIGURE 9:**
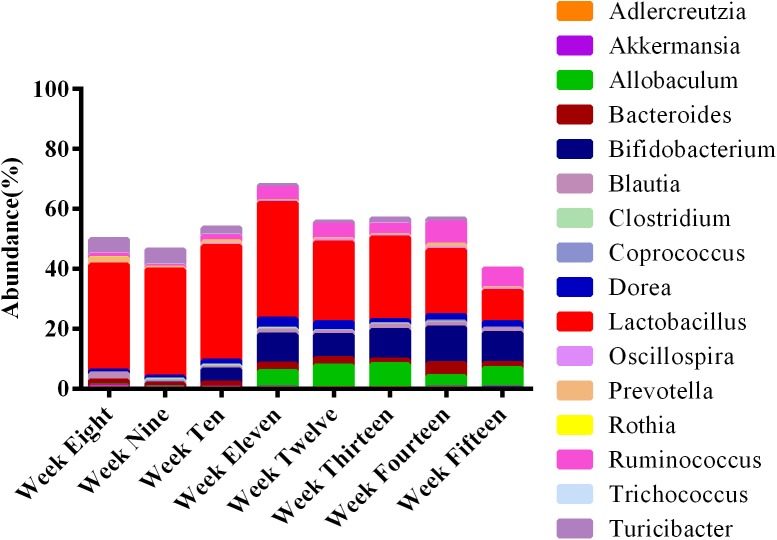
Microbial communities at different stages of diabetes development are dynamic over time. The stacked bar plot reveals that the fecal microbiota was dominated by 16 genera. The average abundance of OTUs per week is represented in bars. *Lactobacillus* was most prominent among the rats at all ages.

Employing the test for equal proportions (using Pearson’s chi-square test statistic), a total of 16 dominant genera (*p* < 0.05) were found in the feces of ZDF rats among the developmental weeks (Figure 9). From this relative abundance OTU plot, it is clear that *Lactobacillus* was the predominant genus at 8 weeks of age, along with the presence of *Turicibacter*, *Adlercreutzia*, *Ruminococcus*, *Bacteroides*, *Coprococcus*, *Prevotella*, *Blautia*, *Allobaculum*, *Oscillospira*, *Dorea*, *Clostridium*, *Bifidobacterium*, *Rothia*, *Akkermansia*, and *Trichococcus*. At 9 weeks of age, *Lactobacillus* was also the dominant genus, and abundance of *Turicibacter* was slightly reduced. At 10 weeks of age, *Lactobacillus* continued to increase, *Turicibacter* decreased, but *Bifidobacterium* was significantly present. At 11 weeks of age, *Lactobacillus* and *Bifidobacterium* became the most abundant genera, *Ruminococcus*, *Dorea*, and *Allobaculum* were significantly present, and *Turicibacter* was greatly reduced. The abundance of *Allobaculum* increased from weeks 11 to 15. At week 12, *Lactobacillus*, *Bifidobacterium*, and *Allobaculum* remained the dominant genera until week 13. At week 14, *Lactobacillus*, *Bifidobacterium*, and *Ruminococcus* were the most abundant genera, and *Bacteroides* abundance was significantly elevated. At week 15, *Bifidobacterium* abundance was significantly elevated and it remained the dominant genus along with *Lactobacillus*, *Ruminococcus*, and *Allobaculum*. In brief, *Lactobacillus* was the most abundant genus in feces during the progression of diabetes in ZDF rats. The abundance of *Turicibacter* decreased from weeks 8 to 15. The abundance of *Allobaculum* increased from weeks 11 to 15. We provide the bar plot for the average abundance of weekly OTUs to clearly visualize the remaining OTUs in Supplementary Figure S3. It can be seen that 16 genera accounted for 50–60% of the total genera present per group. The remaining percentage is occupied by low abundance taxa (*n* = 62).

**FIGURE 10:**
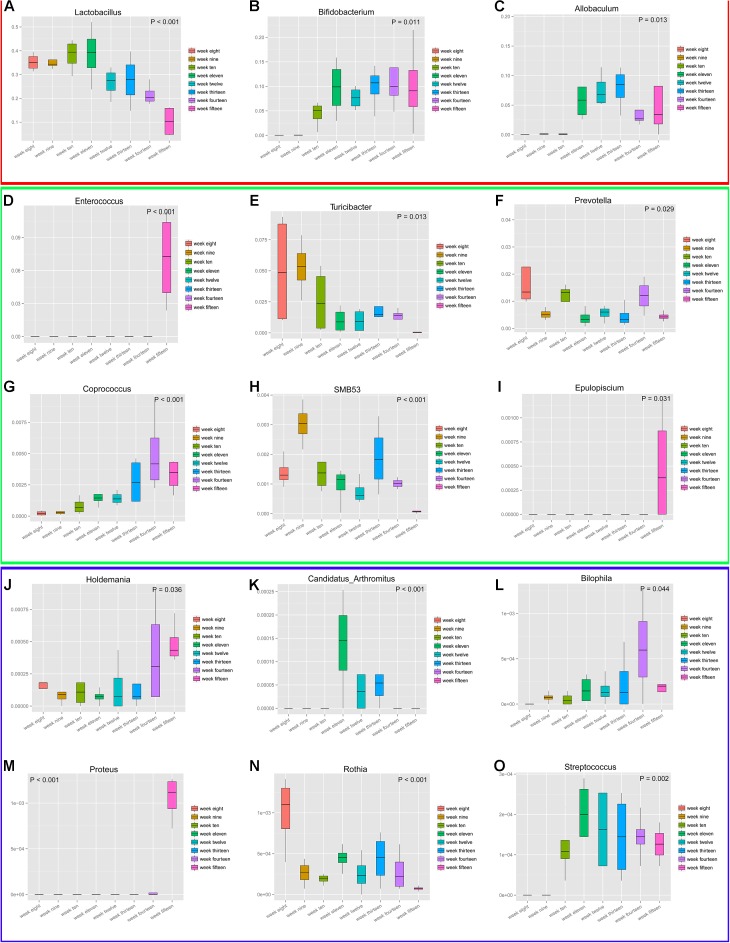
Fifteen genera experienced maximum temporal fluctuations in abundance levels at different stages of diabetes development. These 15 genera **(A–O)** depict highly dynamic variations. *P*-values (*P* < 0.5) are shown on plot corners. Box plots display the following values: the *Y*-axis represents relative abundance of the genus, the *X*-axis represents time grouping; middle box line, represents the median; the upper and lower whiskers represent 1.5 times IQR beyond the upper and lower quartiles, respectively; and dots represent outlier values. Genera *Bilophila*, *Proteus*, *Rothia*, and *Streptococcus* have low abundance levels. For better inspection, these plots have been divided into three parts (red, green, and blue) that reflect their relative abundances.

To analyze the genera with the greatest temporal variation, the relative abundances of species at the genus level were employed. This resulted in the selection of 15 genera based on significant differences (*P* < 0.05) (Figure 10). These data depicting changes in the abundance levels over time indicate that microbial populations changed significantly over time. Although we could analyze the genera that showed large fluctuations in their abundance levels across the developmental weeks, it must be noted that the genera *Bilophila*, *Proteus*, *Rothia*, and *Streptococcus* had significantly low/negligible abundance levels. These fluctuations with low abundance levels might be attributed to sequencing and/or normalization adjustments. The temporal fluctuations of different microbial communities generally indicate that microbial populations are dynamic during the progression of diabetes over time. We speculate that diet, geography, and other environmental factors play an important role in the development of diabetic microbial communities. Finally, the maximum richness in microbial diversity was obtained in rats at 12 weeks of age.

### Physiological Characteristics in ZDF Rats Are Associated With Dysregulated Microbial Taxa

**FIGURE 11:**
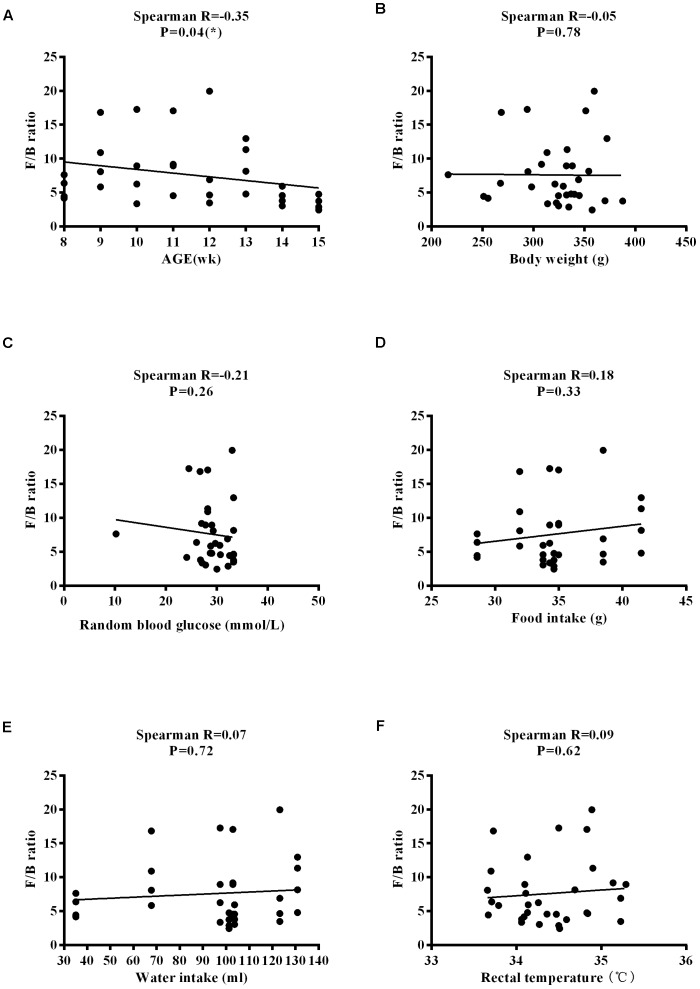
Correlation between the *F*/*B* ratio and physiological characteristics in ZDF rats. **(A)** Age. **(B)** Body weight. **(C)** Random blood glucose. **(D)** Food intake. **(E)** Water intake. **(F)** Rectal temperature.

**FIGURE 12:**
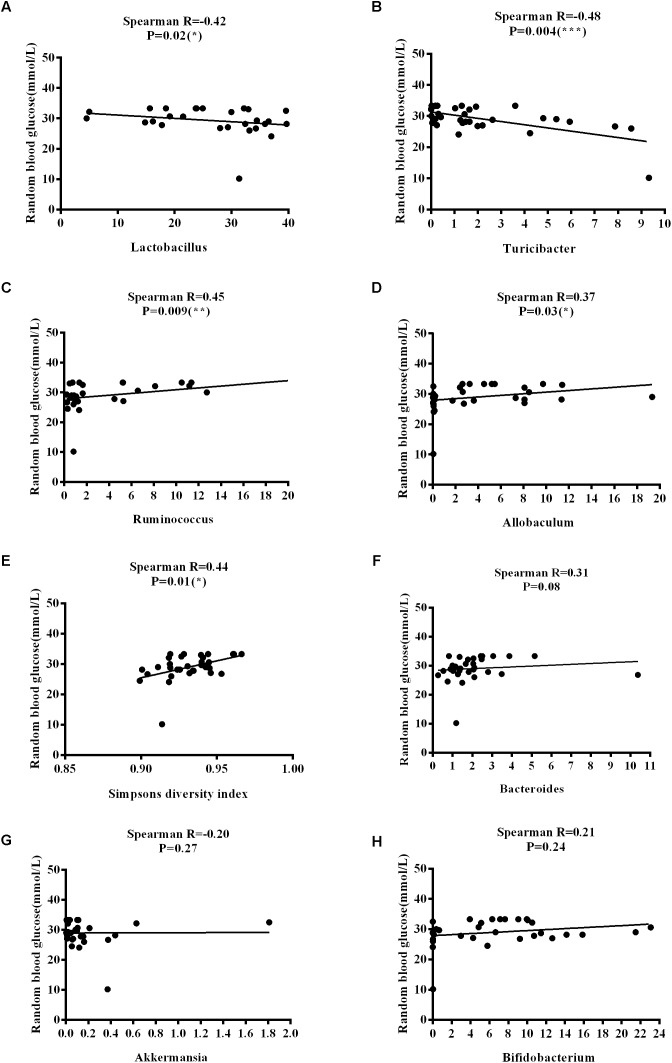
Correlations between random blood glucose levels and variation in microbial communities and Simpson’s diversity index. **(A)** *Lactobacillus*. **(B)** *Turicibacter*. **(C)** *Ruminococcus*. **(D)** *Allobaculum*. **(E)** Simpson’s diversity index. **(F)** *Bacteroides*. **(G)** *Akkermansia*. **(H)** *Bifidobacterium*.

The *Firmicutes*/*Bacteroidetes* (*F*/*B*) ratio is widely used to indicate microbial dysbiosis. Spearman’s correlation analysis showed a significant, negative correlation between F/B and age [*R* = −0.35, *P* = 0.04] (Figure 11A); however, no significant correlations between F/B and body weight, RBG, food intake, water intake, and rectal temperature were found (Figure 11B–F). RBG was strongly and negatively associated with the relative abundance values of *Lactobacillus* [*R* = −0.42, *P* = 0.02] and *Turicibacter* [*R* = −0.48, *P* = 0.004] (Figure 12A,B) but positively associated with the relative abundance values of *Ruminococcus* [*R* = 0.45, *P* = 0.009] and *Allobaculum* [*R* = 0.37, *P* = 0.03] (Figure 12C,D) and SDI [*R* = 0.44, *P* = 0.01] (Figure 12E). We found that there was no significant correlation between RBG and relative abundance values of *Bacteroides* [*R* = 0.31, *P* = 0.08], *Akkermansia* [*R* = −0.20, *P* = 0.27], and *Bifidobacterium* [*R* = 0.21, *P* = 0.24] (Figure 12F–H). The implications of these associations are unclear and would require further experimentation to demonstrate causality.

## Discussion

Microbes play a crucial role in many metabolic-related diseases such as T2DM (Qin et al., 2012). However, systematic studies on the dynamic correlation between microbes and the progression of T2DM are lacking. Therefore, we generated a temporal map of microbial diversity during the progression of T2DM by analyzing the composition of microbes residing in rat feces at different ages. To this end, high-throughput 16S rRNA pyrosequencing was used to study the progressing T2DM fecal microbiome. We used rats of different ages, ranging from 8 to 15 weeks (diabetic stage). We observed that physiological characteristics in ZDF rats, including body weight, food intake, water intake, and RBG increased over time; however, glucose tolerance was impaired and diabetic pathological conditions were aggregated. The phyla *Firmicutes*, *Bacteroidetes*, *Actinobacteria*, and *Proteobacteria* dominated the fecal microbiome during the progression of T2DM. We also demonstrated that *Lactobacillus* and *Turicibacter* are the dominant genera at 8–10 weeks of age, while significant richness and diversity were achieved at 11–12 weeks of age. The maximum diversity was achieved at 12 weeks of age. We believe that these findings significantly improve our understanding of the fecal microbiome during the progression of T2DM.

Advances in high-throughput sequencing have made it possible to analyze temporal variations in microbial communities based on time series and longitudinal studies. Unique ecological observations relating to the dynamics, stability, and diversity of microbial populations are revealed in these studies. At present, research on temporal data is still rare, and published studies have often focused on only a few time points in many subjects (Horie et al., 2017; Liu et al., 2017). Complex interactions among microbiota may either occur between microorganisms and their niche environment or between microbes. These factors may contribute to the temporal dynamics of microbial populations. In this study, we used bioinformatic strategies to characterize the specific aspects in fecal samples from T2DM. We traced dynamic changes in the rat fecal microbiome during the progression of T2DM by using well-established statistical methods, such as hierarchical clustering and PCoA.

One of the most important findings from this research is that microbial diversity in the rats increased gradually from 8 to 12 weeks of age and slightly decreased from 13 to 15 weeks of age with the progression of T2DM. The diversity at various periods of T2DM was measured by sophisticated indices. Thus, in future studies, we will address whether microbial diversity affects the severity or incidence of diabetes. Another important finding of this study was that, based on weighted UniFrac distance, the fecal microbiome from rats of similar ages were grouped together in the cluster analyses. At all ages, four phyla, namely *Firmicutes*, *Bacteroidetes*, *Actinobacteria*, and *Proteobacteria* dominated the fecal microbiome. Importantly, *Firmicutes* and *Bacteroidetes* were the predominant phyla at all ages. This is of note as about 95% of the human intestinal microbial metabolic profile belongs to *Firmicutes* and *Bacteroidetes*, followed by *Actinobacteria* and *Proteobacteria* (Dicksved et al., 2007; Jernberg et al., 2007; Naseer et al., 2014), suggesting that the human and rat microbiomes are identical in composition at the phylum level. In addition, at the genus level, we observed that *Lactobacillus* was the dominant genus at 8 weeks of age and remained predominant throughout T2DM development. Several reports have indicated that an increase in the abundance of *Lactobacillus* is essential for the prevalence of obesity (Ley et al., 2006; Turnbaugh et al., 2006; Million et al., 2012a,b). Similarly, reports also illustrate the presence of a greater number of *Lactobacillus* in patients with T2DM and ZDF rats, which contributes to the development of chronic inflammation of diabetes (Zeuthen et al., 2006; Sato et al., 2014; Gu et al., 2016). Moreover, *Lactobacillus* is involved in insulin resistance (Le et al., 2012) and is coincident with bile salt hydrolase enzymatic activity, thereby disturbing lipid and glucose metabolism and contributing to T2DM (Tremaroli and Backhed, 2012). We observed a slight decrease in the abundance of *Turicibacter*, a Gram-positive, strictly anaerobic bacterium (Bosshard et al., 2002), in rats at 9 weeks of age. It has also been reported that *Turicibacter* was associated with intestinal butyric acid (Zhong et al., 2015). Butyric acid is a short-chain fatty acid that stimulates insulin secretion in the pancreas, increases insulin sensitivity, and alters insulin signaling (Gao et al., 2009; De Vadder et al., 2014). It has significant functions such as providing anti-obesity effects, reducing metabolic stress, and inhibiting inflammatory reactions (Li et al., 2013; Valvassori et al., 2014). However, the metabolism of *Turicibacter* and its interaction with the host in the intestine are still unclear. *Bifidobacterium* was significantly present in rats at 10 weeks of age. *Bifidobacterium*, a dominant member of the intestinal microbiota and probiotic strain of the phylum *Actinobacteria*, was increased in non-diabetics than in T2DM patients. It has been reported that endotoxemia negatively correlates with *Bifidobacterium* and positively correlates with improved glucose tolerance, glucose-induced insulin secretion, decreased endotoxemia, and adipose tissue proinflammatory cytokines (Cani et al., 2007b). This is because *Bifidobacterium* improves mucosal barrier function, thereby decreasing endotoxin levels (Griffiths et al., 2004; Wang et al., 2006). At 11 weeks of age, *Lactobacillus* and *Bifidobacterium* became the dominant genera, and *Ruminococcus* and *Allobaculum* were significantly present. *Ruminococcus* has been shown to assist gut epithelial cells to absorb sugars, which could contribute to weight gain in the host. Nobel et al. (2015) reported that *Allobaculum* was an important functional phenotype of metabolic dysbiosis. Additionally, it has been reported that *Allobaculum* is the abundant genus in mice that are particularly fed on low-fat and high-fat diets (Ravussin et al., 2012). At 12 weeks of age, *Lactobacillus*, *Bifidobacterium*, and *Allobaculum* remained the dominant genera until week 13. Likewise, *Lactobacillus* was also the dominant genus in 12-week-old TSOD mice (12-week-old TSOD mice exhibit typical clinical status of diabetes) (Horie et al., 2017). At 14 weeks of age, *Bacteroides* was significantly elevated. *Bacteroides* is a Gram-negative bacterium that contains lipopolysaccharide in its cell wall (Finegold et al., 2010). It is known that a large number of Gram-negative bacteria in the intestine may damage the gut barrier, releasing lipopolysaccharide into the bloodstream and triggering a low degree of chronic inflammation (Cani et al., 2007a). Although *Lactobacillus* and *Turicibacter* were the predominant genera in 8- to 10-week-old rats, *Bifidobacterium*, *Lactobacillus, Ruminococcus*, and *Allobaculum* were the most abundant genera in 15-week-old rats. One possible reason is that, at the genus level, *Lactobacillus* predominates throughout the progression of T2DM. *Turicibacter* only predominated in the early stage of diabetes in ZDF rats, while the abundance of *Bifidobacterium, Ruminococcus*, and *Allobaculum* increased with the aggravation of the pathological state of diabetes and elevated blood glucose levels in rats (Gu et al., 2016; Kim et al., 2017). Blood glucose levels may also affect the abundance of the bacteria, however, the causal relationship between them is still unclear. Future research needs to prove the relationship between them. In addition, as the rats continued eating high-fat diets, *Allobaculum* may also gradually increase in abundance. These all indicated that, at the genus level, the fecal microbes in the diabetic stage of ZDF rats changed dynamically. Of interest, when compared with previous weeks, a relatively higher diversity was observed at the genus levels at 12 weeks of age, whereas during 13–15 weeks of age, lower diversity was achieved.

Intriguingly, we have observed a gradual decrease in the abundance of *Akkermansia muciniphila* with the progression of diabetes. *A. muciniphila* is an adherent mucin-degrading bacterium that has been proposed to modulate intestinal health, energy balance, and glucose balance (Everard and Cani, 2013). Recent studies uncovered that *A. muciniphila* decreases in prediabetic patients (Yassour et al., 2016; Allinet al., 2018) and has a negative association with T2DM, implying a protective effect on diabetes (Everard et al., 2013; Chen M. et al., 2018; Mithieux, 2018). *A. muciniphila* is found in the feces of rats, and a decrease in its abundance is associated with the progression of diabetes. The mechanisms and factors that play an important role in promoting the growth of the bacteria remain unknown and will play an important role in modulation of metabolic diseases.

The presence of *Ruminococcus* in the stool was a surprising finding. Of interest, *Ruminococcus* was earlier detected in the feces and gut flora from adults with T2DM (Everard and Cani, 2013). Since a number of *Ruminococcus* species are known to be associated with metabolic diseases, the identification of *Ruminococcus* to the species level might be critical for further understanding the relationship between *Ruminococcus* and diabetes and its effect on the development of metabolic diseases.

Notably, we found that the *F*/*B* ratio is negatively correlated with age. The *F*/*B* ratio, the ratio of the two largest microbial phyla, has previously been considered to be a sign of obesity and T2DM (Turnbaugh et al., 2009). However, the causality of this transformation of the phyla as an integral part in the health of the organism, and even as a useful biomarker, has recently been questioned (Brown et al., 2012). RBG is negatively associated with *Lactobacillus* and *Turicibacter*, while it is positively correlated with *Ruminococcus*, *Allobaculum* and SDI, suggesting that under conditions of diabetes, *Lactobacillus* and *Turicibacter* may help recover blood glucose levels. There may be mutual influence between the blood glucose levels and microbial diversity; however, precisely how they are affected and whether there is a link in function and causality requires further proof of experimentation. The lack of statistical significance between the *F*/*B* ratio and body weight, food intake, water intake, and RBG, and the lack of statistical significance between RBG levels and *Akkermansia*, *Bacteroides*, and *Bifidobacterium*, may be due to variability among individuals and a small sample size. Although a few studies have shown a correlation between specific physiological characteristics and specific gut microbes, to the best of our knowledge, this is the first study to attempt to correlate the dynamic physiological characteristics of 8–15-week-old ZDF rats with microbes. Further investigations are required with a greater number of animals or a human cohort to verify the results of this study and to determine the possible underlying mechanisms.

T2DM is a complex metabolic disorder. Beyond the widely-accepted concept that genetic factors play an important role in diabetes susceptibility, growing evidence has demonstrated that environmental factors (such as commensal bacteria, chemicals, diet, and viruses) may also modify diabetes development. Of these factors, the gut microbiota has been shown to play an important role in influencing the progression of T2DM. This has been supported by results from both human research and animal studies, especially the discordant incidence of diabetes in monozygotic twins who are genetically identical (Tai et al., 2015). *Lactobacillus* might be used as one of the genera in experiments, showing a role for the microbiome during the progression in T2DM. We also observed that among groups of rats of different ages, *Firmicutes* and *Bacteroides* are the dominant phyla. These observations confirm findings from patients with diabetes, where these phyla were found in the feces of diabetics (Dicksved et al., 2007; Jernberg et al., 2007; Naseer et al., 2014). In this study, we observed that the microbiome of rats was predominated by the genera *Lactobacillus*, *Turicibacter*, *Bifidobacterium*, *Ruminococcus*, *Allobaculum*, and *Bacteroides.* Studies in humans suggest that the human fecal microbiome is primarily dominated by *Bifidobacterium*, *Bacteroides*, *Escherichia*, *Intestinibacter*, *Prevotella*, *A. muciniphila*, *Blautia*, and *Ruminococcus* (Moreno-Indias et al., 2014; Wu et al., 2017). These data seem to indicate that the rat fecal microbiome has some similarities with the human fecal microbiome while harboring some other genera. This observation indicates that the ZDF rat can be used as a model for studying the T2DM microbiome. Analyzing the changes in the rat fecal microbiome and comparing them with the available data from human clinical studies will be interesting. In addition, increasing evidence indicates miRNAs have close associations with diabetes, so miRNA biomarkers will be particularly useful in early diagnostics of diabetes (Chen et al., 2017; Chen X. et al., 2018; Hu et al., 2018; Zhao et al., 2018a,b). It would be meaningful to correlate miRNA biomarkers with gut microbes in T2DM, but this is still beyond the scope of this study.

Although our research has monitored the changes in microbiome composition and the diversity of feces in ZDF rats with age and disease progression, important questions remain unanswered. These include whether the fecal microbiome is influenced by sex or diet. A number of earlier studies have shown that sex may influence the fecal microbiome (Shastri et al., 2015; Fields et al., 2018). Diet can also affect the intestinal flora, especially high-fat diets (Daniel et al., 2014; Chi et al., 2018; He et al., 2018) Thus, one of the weaknesses of the present study is that we did not take into account the effects of sex or diet on the rat fecal microbiome. Another limitation of this study is that our results are based on a small sample size. Further validation in a larger number of animals or a human cohort is needed. Moreover, we have not demonstrated a causal relationship between microbiota and diabetes. Future work to establish causality would involve the isolation of specific taxa and transfer of anaerobically cultured clones into germ-free animals to demonstrate the development of diabetes in recipient animals. Unfortunately, there was no negative control group included to dissect the specific correlation between microbial changes and disease progression. Future studies should include negative control studies to better understand the correlation between microbial changes and disease progression. The main strength of this research is that fecal microbiome composition was associated with age and the progression of diabetes. Toward this, fecal samples in the rats of different ages were collected and fecal microbiome analysis was performed. To conclude the association between the diabetic microbiome and age and disease progression in rats, we performed rigorous analyses.

This research differs from other research projects which address the effect of the gut microbes on diabetes. In this study, we monitored the changes in the fecal microbiome with the growth and disease progression of T2DM. However, many other confounding factors may affect fecal microbes, including stress and feed type, as in the gut microbiome (Hufeldt et al., 2010). In summary, we have monitored the changes in the fecal microbiome in ZDF rats from 8 to 15 weeks of age by using deep sequencing. This analysis suggests that the microbial composition is associated with the age and progression of diabetes in rats.

## Conclusion

Other research has implicated the microbiome in playing an important role in metabolic diseases such as diabetes. However, there is a lack of time-resolved microbial changes during the progression of diabetes. This understanding is crucial for creating new interventions for curing metabolic diseases such as diabetes. In this study, we monitored changes in the fecal microbiome during the progression of diabetes from 8 to 15 weeks of age. The fecal microbiome in rats was highly dynamic and underwent major changes during the progression of diabetes. The determined time-dependent alteration of the fecal microbiome supports further investigation to determine whether *Lactobacillus*, *Turicibacter*, *Bifidobacterium*, *Allobaculum*, *Ruminococcus*, and *Akkermansia* may play functional roles in the progression of diabetes before any intervention can be considered.

## Author Contributions

LBZ and XL conceived the idea, directed the project, and designed the experiments. HX, WZ, and LJZ performed the experiments, obtained the samples, and acquired the data. WZ analyzed the data and wrote the manuscript. LBZ, XL, and WZ edited the manuscript. All authors read and approved the final manuscript.

## Conflict of Interest Statement

The authors declare that the research was conducted in the absence of any commercial or financial relationships that could be construed as a potential conflict of interest.

## Abbreviations

F/B: *Firmicutes*/*Bacteroidetes*
IQR: interquartile range
OGTT: oral glucose tolerance test
OTUs: operational taxonomic units
PCoA: principal coordinates analysis
RBG: random blood glucose
SDI: Simpson’s diversity index
T2DM: type 2 diabetes mellitus
ZDF: Zucker diabetic fatty
