# Supplementary material for: Dynamic Development of Fecal Microbiome During the Progression of Diabetes Mellitus in Zucker Diabetic Fatty Rats
Source: Front Microbiol. 2019 Feb 14;10:232. doi: 10.3389/fmicb.2019.00232 (PMC6382700; doi:10.3389/fmicb.2019.00232)
Supplement: Supplementary file 1 [file Table_1.DOCX]

Supplementary Material

**Dynamic Development of Fecal Microbiome in Diabetes Mellitus of Zucker Diabetic Fatty Rats**

Wen Zhou, Huiying Xu, Libin Zhan*, Xiaoguang Lu* and Lijing Zhang

*** Correspondence:**
Libin Zhan
zlbnj@njucm.edu.cn
Xiaoguang Lu
dllxg@126.com

# Supplementary Figures and Tables

## Supplementary Figures


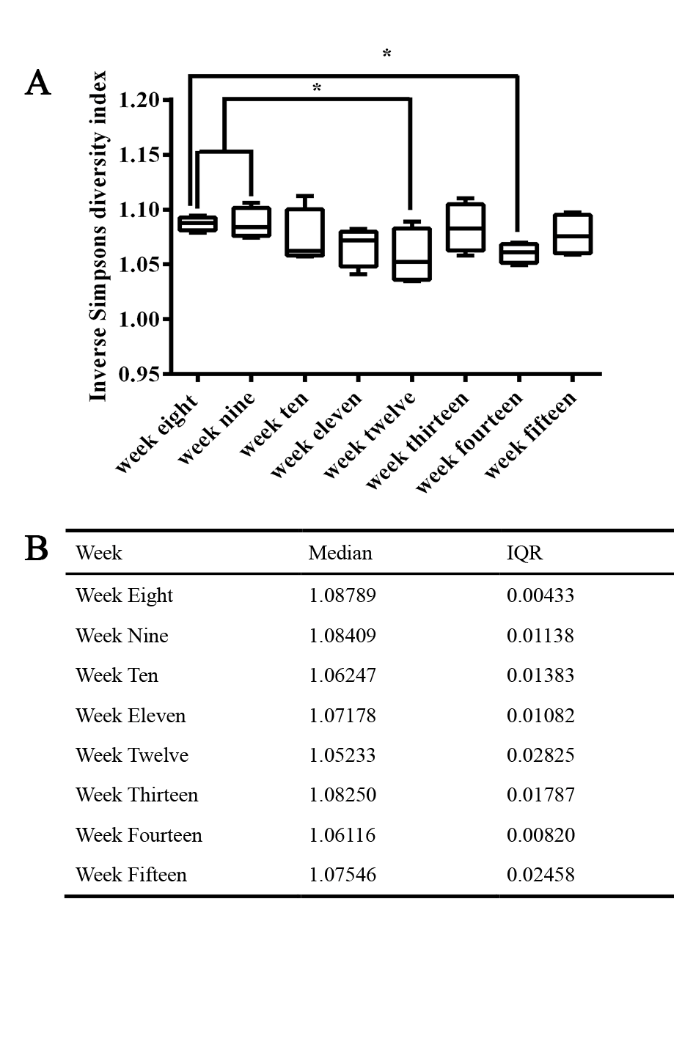


**Supplementary Figure 1.** (**A**) Inverse Simpson’s diversity index follows the same trend as Simpson’s diversity index. (**B**) The table presents the median and interquartile range (IQR) values.





**Supplementary Figure 2.** Mean abundance measure along with the standard error for each phylum.





**Supplementary Figure 3.** Mean abundance measure along with the standard error for each genus.

## Supplementary Tables

**Table S1 The number of fecal samples collected from four ZDF rats**

| S.No | The number of fecal sample | | | | | | | |
| --- | --- | --- | --- | --- | --- | --- | --- | --- |
|  | Week Eight | Week Nine | Week Ten | Week Eleven | Week Twelve | Week Thirteen | Week Fourteen | Week Fifteen |
| ZDF 1 | 1 | 1 | 1 | 1 | 1 | 1 | 1 | 1 |
| ZDF 2 | 1 | 1 | 1 | 1 | 1 | 1 | 1 | 1 |
| ZDF 3 | 1 | 1 | 1 | 1 | 1 | 1 | 1 | 1 |
| ZDF 4 | 1 | 1 | 1 | 1 | 1 | 1 | 1 | 1 |
| Count | 4 | 4 | 4 | 4 | 4 | 4 | 4 | 4 |
| Sum | 32 | | | | | | | |

**Table S2 Median and interquartile range (IQR) values for Figure 1**

| Week | Median | IQR |
| --- | --- | --- |
| Week Eight | 0.91921 | 0.00511 |
| Week Nine | 0.92244 | 0.01346 |
| Week Ten | 0.94121 | 0.01383 |
| Week Eleven | 0.93303 | 0.01222 |
| Week Twelve | 0.95040 | 0.03151 |
| Week Thirteen | 0.92381 | 0.02097 |
| Week Fourteen | 0.94237 | 0.00922 |
| Week Fifteen | 0.92996 | 0.02848 |

**Table S3 Number of replicates per week and reads mapping to each sample**

| S.No | Week Eight | Week Nine | Week Ten | Week Eleven | Week Twelve | Week Thirteen | Week Fourteen | Week Fifteen |
| --- | --- | --- | --- | --- | --- | --- | --- | --- |
| 1 | 59446 | 72109 | 65623 | 65075 | 49406 | 69541 | 48381 | 54114 |
| 2 | 55928 | 86407 | 59020 | 62288 | 47234 | 67571 | 42528 | 65239 |
| 3 | 58553 | 82755 | 61712 | 58678 | 50892 | 69287 | 50004 | 54828 |
| 4 | 66726 | 87541 | 70500 | 61810 | 46554 | 61109 | 47758 | 45809 |
| Count | 4 | 4 | 4 | 4 | 4 | 4 | 4 | 4 |
| Average | 60163 | 82203 | 64214 | 61963 | 48522 | 66877 | 47168 | 54998 |
| Sum | 240653 | 328812 | 256855 | 247851 | 194086 | 267508 | 188671 | 219990 |
| Total reads | 1944426 |  |  |  |  |  |  |  |
